# Technology-assisted stroke rehabilitation in Mexico: a pilot randomized trial comparing traditional therapy to circuit training in a Robot/technology-assisted therapy gym

**DOI:** 10.1186/s12984-016-0190-1

**Published:** 2016-09-15

**Authors:** Karla Bustamante Valles, Sandra Montes, Maria de Jesus Madrigal, Adan Burciaga, María Elena Martínez, Michelle J. Johnson

**Affiliations:** 1 Department of Physical Medicine and Rehabilitation, University of Pennsylvania, Philadelphia, PA USA; 2 Department of Biomedical Engineering, University of Pennsylvania, Philadelphia, PA USA; 3 Orthopaedic and Rehabilitation Engineering Center (OREC), Marquette University, Milwaukee, WI USA; 4 Biomedical Engineering, ITESM, Campus Chihuahua, Chihuahua, Chihuahua Mexico; 5 Centro de Rehabilitacion y Educacion Especial, DIF, Chihuahua, Chihuahua Mexico

**Keywords:** Stroke, Rehabilitation, Robot therapy, Affordable, Low-and-Middle Income Countries (LMIC), Upper limb, Lower limb, Global Health

## Abstract

**Background:**

Stroke rehabilitation in low- and middle-income countries, such as Mexico, is often hampered by lack of clinical resources and funding. To provide a cost-effective solution for comprehensive post-stroke rehabilitation that can alleviate the need for one-on-one physical or occupational therapy, in lower and upper extremities, we proposed and implemented a technology-assisted rehabilitation gymnasium in Chihuahua, Mexico. The Gymnasium for Robotic Rehabilitation (Robot Gym) consisted of low- and high-tech systems for upper and lower limb rehabilitation. Our hypothesis is that the Robot Gym can provide a cost- and labor-efficient alternative for post-stroke rehabilitation, while being more or as effective as traditional physical and occupational therapy approaches.

**Methods:**

A typical group of stroke patients was randomly allocated to an intervention (*n* = 10) or a control group (*n* = 10). The intervention group received rehabilitation using the devices in the Robot Gym, whereas the control group (*n* = 10) received time-matched standard care. All of the study subjects were subjected to 24 two-hour therapy sessions over a period of 6 to 8 weeks. Several clinical assessments tests for upper and lower extremities were used to evaluate motor function pre- and post-intervention. A cost analysis was done to compare the cost effectiveness for both therapies.

**Results:**

No significant differences were observed when comparing the results of the pre-intervention Mini-mental, Brunnstrom Test, and Geriatric Depression Scale Test, showing that both groups were functionally similar prior to the intervention. Although, both training groups were functionally equivalent, they had a significant age difference. The results of all of the upper extremity tests showed an improvement in function in both groups with no statistically significant differences between the groups. The Fugl-Meyer and the 10 Meters Walk lower extremity tests showed greater improvement in the intervention group compared to the control group. On the Time Up and Go Test, no statistically significant differences were observed pre- and post-intervention when comparing the control and the intervention groups. For the 6 Minute Walk Test, both groups presented a statistically significant difference pre- and post-intervention, showing progress in their performance. The robot gym therapy was more cost-effective than the traditional one-to-one therapy used during this study in that it enabled therapist to train up to 1.5 to 6 times more patients for the approximately same cost in the long term.

**Conclusions:**

The results of this study showed that the patients that received therapy using the Robot Gym had enhanced functionality in the upper extremity tests similar to patients in the control group. In the lower extremity tests, the intervention patients showed more improvement than those subjected to traditional therapy. These results support that the Robot Gym can be as effective as traditional therapy for stroke patients, presenting a more cost- and labor-efficient option for countries with scarce clinical resources and funding.

**Trial registration:**

ISRCTN98578807.

## Background

A stroke is a cerebrovascular accident provoked by an interruption of the blood supply to the brain or a rupture of a blood vessel in the brain. Stroke is one of the leading causes of death worldwide, with over two-thirds of stroke deaths occurring in developing countries [1, 2]. According to the World Health Organization, approximately 15 million people suffer a stroke each year [3]. In the United States (US), 610,000 new cases are reported yearly [4]. Stroke rates are expected to increase in the next 40 years as the aging population in the US expands [5]. In Mexico, stroke is a major public health issue [6]. It is the 3^rd^ leading cause of death among people aged sixty years or older and the fifth leading cause of death in people aged 15 to 59 years old [7, 8]. It has been projected that cerebrovascular disease in middle income countries will be the leading cause of burden of disease by 2030 [9].

Stroke is one of the leading causes of disability in both developing and developed countries [10]. Common disabilities after stroke include: hemiparesis, motor deficits, cognitive deficits, aphasia, proprioceptive deficits and depressive symptoms [11, 12]. Typically in high-income countries as the USA, after a stroke, one-third of the patients are left with a permanent disability that can range from a mild to a severe, long-term disability [3, 11, 13], while in low-and middle income countries (LMICs) those with disability will often be more severe with higher long term economic burden and impact on society [14, 15]. The economic burden of stroke is usually classified in terms of medical expenses and indirect costs due to loss of productivity. It has been estimated that medical and indirect costs in the US from 2012 to 2030 will increase from 71.55 to 183.13 billion and 33.54 to 183.13 billion, respectively [16]. While the cost of medication and therapy in developing countries may be lower than in developed countries, the average family income in developing countries is also significantly lower. For example, the family income of more than 65 % of population in Mexico is around 250 USD a month, which is insufficient to cover the treatment of hypertension for just one family member [17]. Another significant problem in developing countries is the lack of trained healthcare personnel, especially physical therapists in rehabilitation settings. Mexico has 1,500 physical therapists for a population of 125,000,000 while the ratio of health workers is about 2.85 personnel per 1000 patients in Mexico compared to 14.66 per 1000 patients in the US [18]. In addition to the low numbers of therapists and health workers, the cost of medical equipment, which includes rehabilitation technology, is higher in LMICs. These higher costs are due to the minimum amount of research and development of such equipment that takes place in LMICs and the need to import foreign medical equipment with marked up cost incorporating importation taxes and third party distribution profits [16]. Short supply of trained healthcare physicians and medical technologies makes access to rehabilitation facilities and associated services limited and not affordable to a large part of the population [19, 20].

Inpatient and outpatient stroke rehabilitation is recommended and is associated with better functional outcomes after stroke. A wide range of post-stroke rehabilitation therapies exist to address motor impairments and to ultimately improve function and independence in the community [21, 22]. Langhorne et al. systematically reviewed interventions for motor impairment rehabilitation after stroke, which include: mixed approach, motor learning, neurophysiological approaches, bilateral training, biofeedback, constraint induced movement therapy, and robotics, among others [23]. This study concluded that the most promising intervention for upper-limb function seems to be constraint-induced movement therapy for the upper limb and fitness training, high intensity therapy, and repetitive task training for gait improvement. In contrast, Pollock et al. concluded that there is not enough evidence to support that one post-stroke rehabilitation therapy is more effective than the others and suggests that a mix of different rehabilitation therapies may be more effective to improve function and mobility. In addition, therapies with consistent engagement in 30 to 60 min sessions 5 to 7 days a week were found to have better outcomes, regardless of the therapies used [24]. Comprehensive, intensive, and task-specific therapies are thought to be the most likely to produce therapeutic effects on stroke patients [25]. Many of the above strategies are standard of care in high-income countries, but sparse rehabilitation infrastructure and low numbers of rehabilitation clinicians, make applying these recommendations difficult in LMICs.

Evidence supports rehabilitation robots’ role in improving rehabilitation outcomes and reducing barriers to rehabilitation services [26–32]. Rehabilitation Robots have the potential to narrow the gap between those who recover and those who do not. Robots can improve functional outcomes, increase access and time of therapy, and maximize resources in a variety of rehabilitation environments [28, 29, 31]. Examples of these systems include the Lokomat (Hocoma, Inc) for lower limb gait therapy [30] and ARMin [33], MAHI Exo II [34], T-WREX [35] precursor of the commercial Armeo Spring (Hocoma, Inc) and InMotion Robots (Interactive Motion Technologies) [27, 32] for upper limb therapy. They are characterized by the use of a multi-degree of freedom robot manipulator for training the impaired arm of the patient and guiding the person through therapeutic exercises ranging from point-to-point reaching to real activities. Forces are applied as needed to assist the patients to move when they are unable to move independently or self-correct their movement. Meta-analyses and clinical trials show that rehabilitation outcomes with robots are comparable to standard and intensity-matched stroke rehabilitation and significantly improve functional outcomes in terms of motor control and ADL function [28, 29]. The use of robotic therapy has also been proven to be promising in its potential cost savings and efficient use of the therapists’ working time, throughout the labor-intensive and long-term process of stroke rehabilitation [36, 37]. Wagner and colleagues suggest that after 36 weeks, the total costs of robot therapy were comparable for the three groups they trained “($17,831 for robot therapy, $19,746 for intensive comparison therapy, and $19, 098 for usual care)” [32]. Hesse and colleagues demonstrate that robot circuit training may ultimately result in a 50 % savings over individualized training (cost per treatment session were 4.15 Euro versus 10.00 Euro) [26].

Most robot-assisted therapy devices are expensive with cost greater than 50,000 to 100,000 USD for a single unit [32]. However, more recent affordable systems are being proposed for the home and rehabilitation facilities use such as Reha-Stim line of devices [38, 39], TyroMotion’s Pablo® [40], Hand Mentor Pro™ from Kinetic Muscle, Inc. [41], and Haptic Knob [42] among others. In Germany, a suite of simple robots arranged in the ARM Studio was able to improve functional outcomes after stroke for patients in an inpatient rehabilitation center [43]. In the USA, a suite of simple computer/robot technologies was able to assess and improve functional outcomes of the upper limb [44]. These studies suggest that affordable technology solutions for rehabilitation may provide the means to bridge the gap between the scarcity of rehabilitation providers and their growing disabled population in LMICs. Very few studies have critically examined the issue of accessible post-stroke rehabilitation and have attempted to implement cost-efficient rehabilitation alternatives in LMICs, such as Mexico. Hence, the goal of this study was to develop and deliver an effective and cost and labor efficient method of post-stroke rehabilitation that encouraged continued rehabilitation and was more or as effective as traditional physical and occupational therapy approaches.

This paper describes a pilot randomized controlled trial where patients were allocated to receive traditional therapy (control) or Robot Gym therapy. We report pre- and post-clinical results on assessment tests for the upper and lower limbs after 24 sessions of therapy with the Gym. The Robot Gym created an affordable stroke therapy clinic utilizing the TheraDrive robot [45–47] and other similar low-cost technologies in combination with game therapy. This post-stroke treatment concept is unique in that it combines a wide-range of assistive and robot technologies. In addition, it supports that a viable, accessible rehabilitation solution can include affordable robots along with other cost-efficient rehabilitation technologies.

## Methods

### Protocol description

The goal of this pilot study is to evaluate the effectiveness of a stroke intervention protocol implemented via a low-cost robotic gym (Robot Gym) used in under-supervised conditions. In this parallel designed protocol, a typical group of chronic stroke patients were randomized to receive rehabilitation using the devices in the rehabilitation robot suite (intervention group; *n* = 10) or to receive time-matched standard care (control group; *n* = 10) at the Rehabilitation Center in Chihuahua, Mexico (CREE, *Centro de Rehabilitacion y Educacion Especial*). Patients were consented and then evaluated by clinicians to assess motor impairment in the upper and lower limbs. As standard of care, all of the patients at this center go through psychological evaluations; these medical charts were requested to help determine eligibility. The sample size for this pilot study was 12 patients per group, a convenient sample size allowing for additional 25 % patients to account for drop out, resulting in a total of 30 subjects. It was foreseen that recruitment could be a possible problem due to inaccurate or missing patient’s clinical history records.

Potential subjects were interviewed and prescreened by rehabilitation physicians. If a patient was eligible, he was invited to participate by the physician. Patients interested in participating in this protocol subsequently met with the principal investigator and the purpose of this study was explained. All patients were informed about the potential risks that could be presented during the study, which include: frustration in being unable to complete some steering tasks, fatigue, discomfort due the equipment position, muscle soreness in legs and arms due to exercise, irritation or pressure sores due the use of FES, allergic reactions to the electrodes, and a swelling of the extremities involved on the exercises. All study participants gave informed consent prior to formal enrollment in the protocol and were assigned a consecutive number as they were admitted. For example, the first patient enrolled was assigned number P1; the following patients were assigned consecutive numbers according to their recruitment (P2, P3, P4…). An allocation sequence was generated using the Epidat 4.0 software to randomly assign numbers from 1 to 30 into two groups: the control group (CT) or the robotic or intervention group (RT) [48]. All study participants signed an informed consent and a privacy form. The protocol, the recruitment form, authorization for release of protected health information (PHI), and the informed consent with the registration number HR-2110, were reviewed and approved by the Marquette University Institutional Review Board (IRB) and the CREE ethics committee.

### Subject population

All patients assessed for eligibility were clinically diagnosed with hemiplegia from a stroke occurred more than 6 months prior the study. Prior to the study, clinical evaluations were performed by certified clinicians and physiatrist on all subjects to determine their impairment in their extremities, their functional disability, and their cognitive, and depression levels. All of the subjects were included in this study if they were 1) between 21 and 75 years; 2) had hemiparesis due to a cerebral vascular accident (CVA) stroke (confirmed by a physician); 3) were at least 6-months post-stroke and medically stable; 4) had the ability to sit for 60 min and to stand, assisted or unassisted, for 30–40 min; 5) had a score less than eight in the Geriatric Depression Scale [49] indicating mild depression and a likelihood of completing the 24 sessions required; 6) were no be more than moderately cognitively impaired as defined by a Mini-mental Test [41] score greater than 20--they were able to give consent and understand instructions; 7) had residual movement in shoulder flexion/adduction and active elbow flexion/extension and/or residual movement in leg flexion/extension and hip adduction as defined by a Brunnstrom Test Score [39] ranging from 2 to 5; and 8) had a muscle strength scores on the Manual Muscle Test [50] between >1 and <3 in both extremities. All of the subjects included in this study passed the inclusion criteria above. They had their left side affected and had low and medium impairment.

Subjects were excluded if they 1) had excessive spasticity in upper and lower limbs as measured by the Ashworth scale [51] over 4; 2) had pain exceeding 4 on a visual analog pain scale [52, 53]; 3) had total paralysis or muscular contractures of upper or lower extremity; 4) had a history of psychiatric disorder or cardiac problems; 5) had metallic implants near electrical stimulation site or cardiac defibrillators implants; 6) were pregnant or breast feeding; and 7) they were unwilling to participate or comply with the protocol.

### Interventions

All study participants were subjected to 24 two-hour therapy sessions over a period of 6 to 8 weeks.

The CT group received standard rehabilitation therapy, which includes personalized physical and occupational therapy usually in a one-on-one therapist to patient ratio. Standard rehabilitation therapy includes manual mobilizations, heat, ultrasound, therapeutic TENs (transcutaneous electrical nerve stimulation), and repetitive tasks for occupational therapy using tools such as balls, cone sets, exercise bands, among others. All of the patients repeated the clinical evaluations post-therapy. It is important to note that the control group here experienced the standard of care for 2 hours in order to time-match the therapy given to the robot group. Typically patients at CREE would experience only 30 min of the standard of care therapy.

**Fig. 1:**
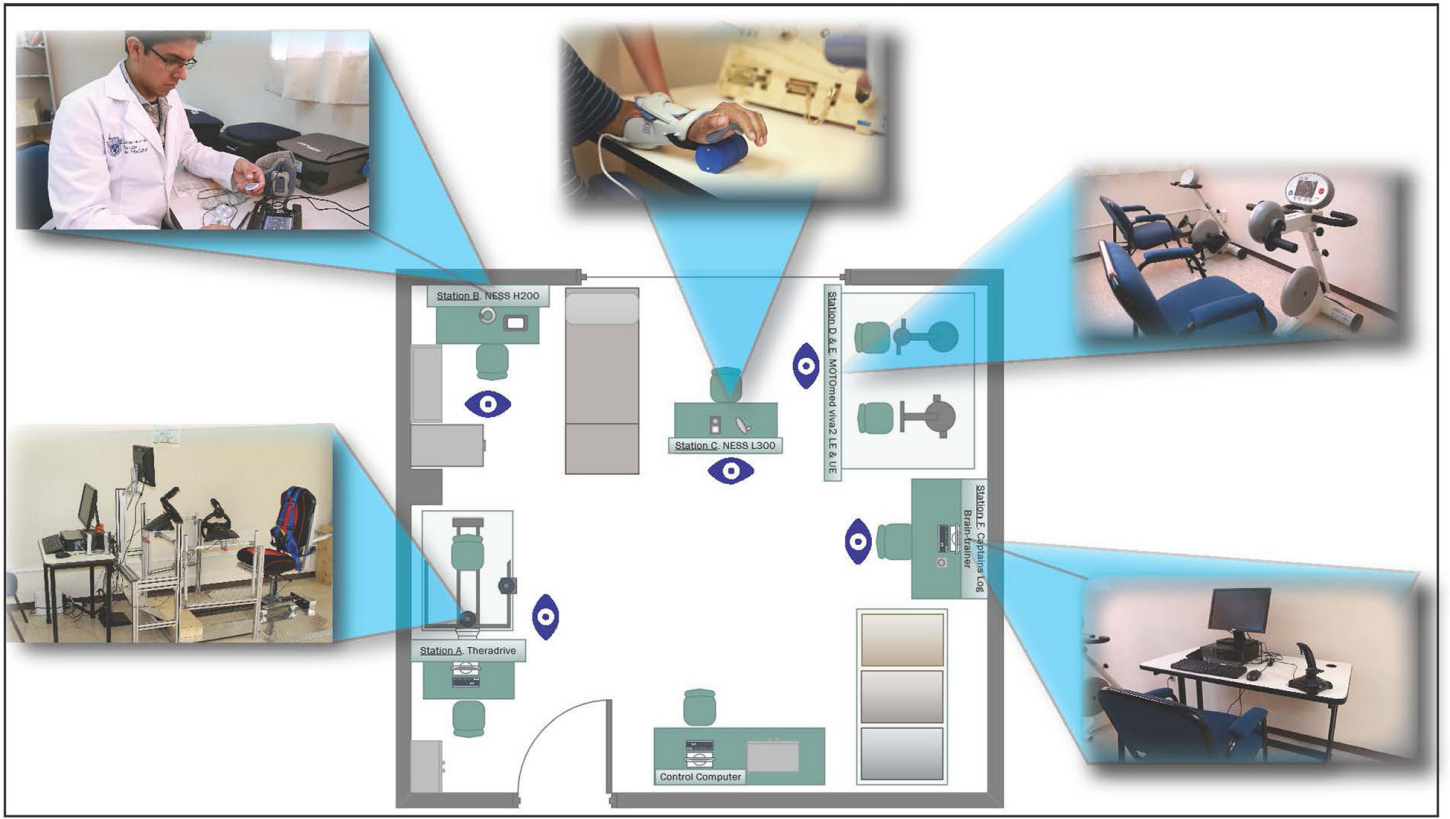
Distribution of the stations inside facility at CREE Chihuahua. Beginning by the door, clockwise, is: the Theradrive system in the first station; the Ness for upper extremity in the second station; the Ness for lower extremity in the third station; the Motomed Viva 2 for upper extremities in the fourth station; the Motomed Viva 2 for lower extremities in the fifth station; and Capitain’s Log Brain-trainer in the sixth station

Robot Gym therapy consists of six stations of computer and motor assisted devices to aid in the motor rehabilitation of the upper and lower extremities, as well as cognitive improvement. The stations are distributed based on a closed circuit structure (Fig. 1), so that when the patient finishes an activity in a station, they can go to the next station without having to walk a long distance. Subjects in the RT group switched stations in the Robot Gym every half hour, working on four stations per day throughout the 24 sessions in order to achieve an equal number of therapy sessions in each station. The following describes the stations.

Station A. TheraDrive: A low cost system for personalized arm rehabilitation based on the theradrive robot was developed [46]. Theradrive targets upper limb dynamic exercises aimed at strengthening and ranging the elbow and shoulder. This device allows patients to play custom-made games and commercial videogames with a Logitech wheel. The wheel can be placed in front of the person or sideways at different angles and heights. For this protocol, the wheel was mounted in front of the person at a 20° angle and centered on the person’s midline. The wheel’s height was adjustable by 15 cm so that the height would be placed for each patient’s comfort. The force-feedback settings on the wheel can be programed to help or disrupt the movement in the upper limbs. These force settings were adapted according to the patient’s initial functional level, i.e., assistive forces were set if the patient was lower functioning, which meant the patient scored lower than 23 points on the Fugl Meyer upper extremity test. Assistive forces decreased and disruptive forces increased as the patient performance improved. The purpose of these settings was to keep patient working at a level that required effort to improve his functionality but to avoid frustration.

Station B and C. NESS H200 and NESS L300: These Bioness devices rehabilitate hand function and foot function, respectively. The NESS H200 (Station B) is a commercial hand rehabilitation system that provides functional electrical stimulation (FES) to help open and close a patient hand while performing functional daily activities as grab, pile and move different objects [54]. The NESS L300 (Station C) is a commercial foot-drop system using FES to assist dorsiflexion to help improve the patient’s gait [54, 55]. Both devices have the capacity to improve voluntary movement of the injured limbs and help with the reeducation of the muscles generating neuroplasticity. The levels of intensity used during electrical stimulation vary between level 5 to 9 and was dependent on the patient tolerance and response to stimulation. The level was adjusted to achieve dorsiflexion without causing intolerable discomfort sensation due to the stimulation.

Station D and E. MOTOmed viva2 LE and MOTOmed viva 2 UE: These MOTOmed devices rehabilitate the lower and upper limbs, respectively. The MOTOmed viva2 LE (Station D) is a commercial lower extremity motor assisted device that allows passive or active resistance training through a series of simple games similar to bicycling [56]. The MOTOmed viva 2 UE is a commercial upper extremity motor assisted device that allows passive or active resistance training through a series of simple games for movement therapy [57]. Both of these devices provide individualized options and biofeedback which are determined by the physician and therapist according to each patient’s abilities. During the study, the main purpose of these devices was to improve the symmetry of upper and lower extremities and motivate the patient to involve their affected limb in the tasks. Their system provides real time feedback about the usage percentage of left and right limb respectively, with the maximum level of resistance tolerated by the patient.

Station F. Captains Log Brain Trainer: This commercial Cognitive Rehabilitation Therapy System provides systematic brain training to patients with brain injury and aims to improve neuroplasticity. It focuses on training different cognitive domains such as memory, attention, perception, reasoning, planning, judgment, general learning and overall executive functioning through computer games. This program has three levels of difficulty (silver, gold and diamond) and an initial test is given to the patient to assess the cognitive state of the patient and assign an initial level of difficulty. The software automatically will increase or decrease difficulty according to patient performance. The Log Brain trainer was adapted to a joystick to allow patients with decreased hand control to manipulate the interface and to promote work on motor visual skills [58].

### Clinical assessments

Upper and lower limb assessments were conducted before and after the 24 sessions of therapy. The Fugl-Meyer Upper Extremity Test (UE-FM) [59–61], the Rancho Los Amigos Functional Test for the upper extremity (UE-FT) [62], and the Box and Block Test (BBT) [63] were used for upper limb assessments. The UE-FM measures several parameters of upper limb function in stroke patients. The physiatrist measured reflex ability, speed, coordination, flexion, and extension synergy through various exercises. These exercises were scored from 0 to 2, where 0 is an exercise that could not be performed, 1 is an exercise that was partially done, and 2 is that the exercise performed without failure. The maximum score a patient could achieve is 66-- the higher the score, the lower the degree of impairment [59–61]. The UE-FT evaluates the integrated function of the total upper extremity in adult patients with hemiparesis. It is arranged in seven levels and administered in approximately 30 min. The higher the level, the lower the degree of impairment [62]. In the BBT, the patient is asked to move wooden cubes from one side of a divided box to the other. If the right limb is analyzed, the patient must move the cube from right to left and vice versa. The objective is to measure patient’s grip when holding the cube, lifting the arm, and crossing through the division in the box to place the cube on the other side [63].

The Fugl-Meyer Lower Extremity Test (LE-FM) [64–66], the 6-minute Walk Test (6MinWT) [67, 68], the 10-meter Walk Test (10MtsWT) [66], and the Timed Up and Go Test (TUG) [69] were used for lower limb assessments. The LE-FM is similar to its counterpart for the upper limb; various parameters of lower limb function in a post-stroke hemiplegic patient are measured. The physiatrist measured lower limb reflex ability, speed, coordination, flexion, and extension synergy through various exercises. The maximum score is 34--the higher the score, the lower the degree of impairment [64–66]. The 6MinWT measures the distance the patient can walk in 6 min. A longer distance walked in the final test compared to the initial test indicates an improvement [67, 68]. The purpose of this test is to assess functional exercise capacity or aerobic capacity endurance. The 10MtsWT measures the time it takes the patient walk 10 m. A shorter time in the final test compared to the initial test indicates an improvement [66]. The objective of this test is to evaluate functional mobility. The TUG measures the time it takes a patient to get up and walk 3 m beginning at a seated position. A shorter time to execute the task in the final test compared to the initial test indicates improvement [69].

### Data analysis and statistics

Clinical assessment data was collected before and after the 24 sessions of therapy. The assessments were divided into qualitative and quantitative assessments. The quantitative tests included: the Fugl-Meyer Upper and Lower Extremity Tests, the 6-minute Walk Test, the 10-meter Walk Test, the Time Up and Go Test, and the Box and Blocks Test. The qualitative test is the Functional Test.

Subjects were divided into therapy groups, Robot Gym (intervention group, RT) and traditional therapy (control therapy, CT). The pre- and post-assessments of each group were compared to determine if there were changes after the 24 sessions of therapy. Quantitative assessments were analyzed using a Student’s t test; a level of *p*-value of ≤0.05 was considered statistically significant. The Anderson-Darling normality test to verify Gaussian distribution was performed. Bartlett test was performed to verify the equality of the variances between the groups. For *p*-values >0.05 in the Bartlett test or the Anderson-Darling test, we considered the *p*-value of the Student’s t parametric test. If this test showed no homogeneity and unequal variance, i.e., *p*-values ≤0.05 in either test, the Kruskal-Wallis non-parametric test was used. We also calculated the effect size for the RT and CT intervention using the Cohen d value [70]. Qualitative tests were analyzed using Single Table Analysis; a level of *p*-value of ≤0.05 was considered statistically significant.

### Therapies cost-effectiveness analysis

To evaluate the cost-effectiveness of the robot gym with respect to traditional therapy, we used the formula reported by Hesse and colleagues (see Eq. 1) to calculate the cost of therapy per session [26]. Equation 1 calculates therapy cost per session by dividing the total number of patients treated by a therapist in a year into the treatment operating expenses, which is the sum of the one-time equipment cost. the yearly maintenance cost of the equipment and the yearly cost of employing the therapist(s) per year [26]. The labor efficiency was taken to be the ratio of therapist needed to patient treated per year.

1 $$ Therapy\  Cost/ Session=\frac{Equipment\  and\  Mainteinance\  Cost s+ Therapist\hbox{'}s\  Annual\  Salary}{\#\  of\  patients\  treated\  in\ a\  year} $$

The prices of the robotic equipment were taken at the time of purchase and the maintenance cost was estimated to be about 25 % of original cost. The staff cost was based on the therapist salaries reported by the Mexican health minister (Secretaría de Salud) [71]. The Stroke guidelines, which is published by the Minister of Health in Mexico, recommends therapy sessions of 3 to 7 days a week, from 20 to 60 min per day, with a rehabilitation specialized therapist and there are no written recommendations for group therapies. The CREE has 278 working days in a year. The mean number of patients that can be treated by one therapist in 1 year for the standard of care was reported by the lead therapist at CREE. This treatment number was calculated two ways for traditional therapy: 1) based on therapy session duration experienced by the control group in the protocol, which was two hours in order to time-match the therapy given to the robot group and 2) based on the session duration typically experience by patients at CREE, which is 30 min of the standard of care therapy. Since our study took place in Mexico, the cost analysis was made in Mexican pesos but we included an approximation of US dollars considering the mean exchange rate in 2011.

## Results

**Fig. 2:**
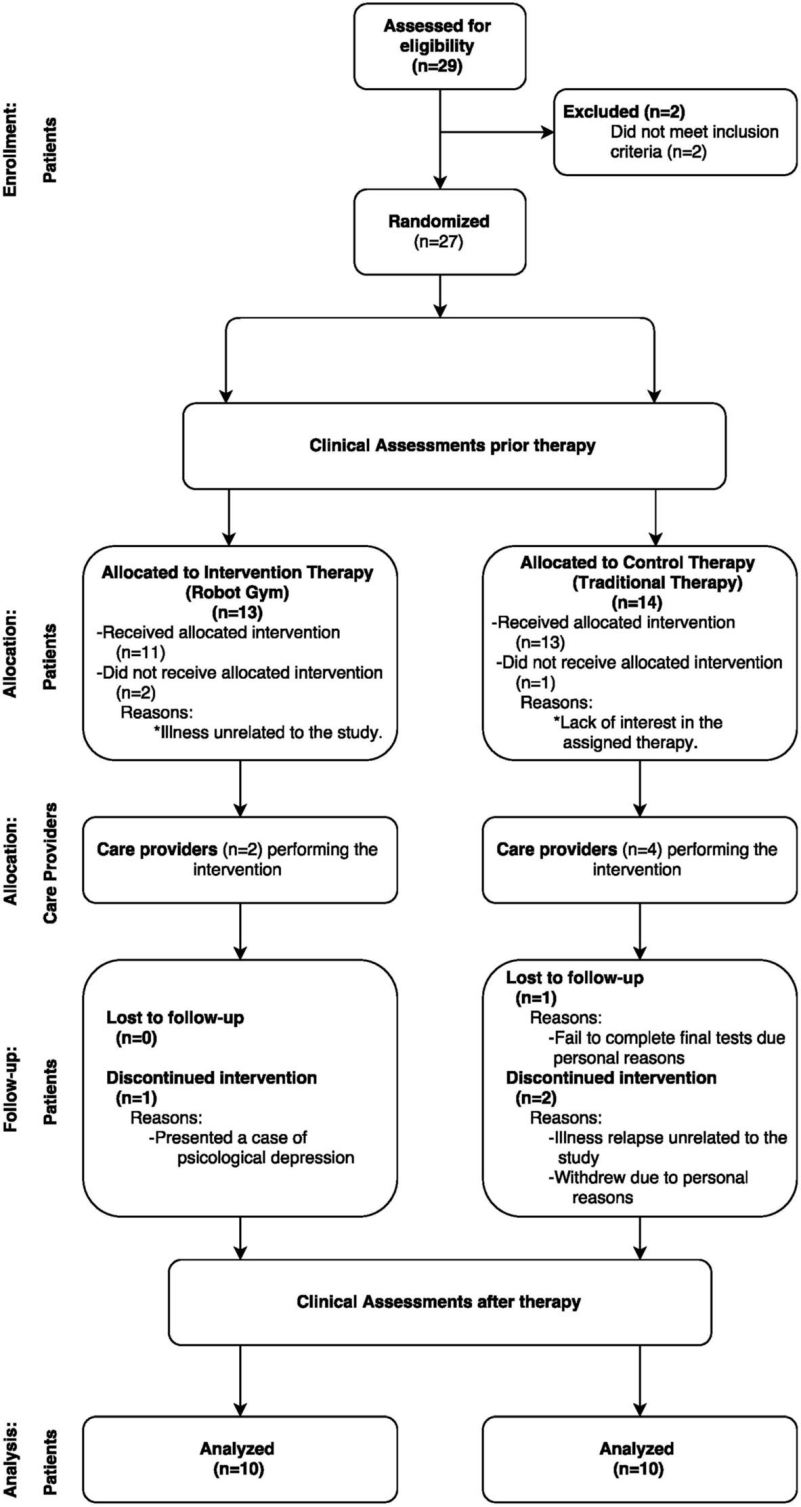
Flow diagram of study participants. This diagram shows the participant flow since recruitment until the information analyses. It also includes the care providers who provided the assigned therapy to each group. Based on recommended flow diagram by CONSORT Group on Nonpharmacologic Treatments [85]

The recruitment period lasted from January 2012 to September 2013. The participant flow is described in Fig. 2. Twenty-seven patients were recruited. Seven patients left the protocol due to different reasons, including the presence or relapse of an illness unrelated to the study, lack of interest in the assigned therapy, and personal reasons. Table 1 presents a summary of the subject demographics.

**Table 1:** Subject pre-intervention information

Patient	Age	Sex	Group	Brunnstrom level	Mini-mental state examination	Geriatric depression scale	Fugl Meyer upper extremity	Fugl Meyer lower extremity
Robot Gym Group								
*P3*	59	M	RT	4	28	3	33	31
*P4*	44	F	RT	3	29	3	23	12
*P5*	52	M	RT	5	23	1	51	27
*P6*	40	F	RT	2	29	7	21	28
*P7*	25	F	RT	4	30	3	30	30
*P9*	29	F	RT	2	29	4	9	21
*P10*	33	F	RT	3	29	0	19	23
*P12*	44	M	RT	3	29	5	17	21
*P13*	62	F	RT	3	29	1	8	14
*P17*	53	F	RT	3	30	2	19	24
*Control Group*								
*P8*	71	F	CT	2	28	1	15	15
*P11*	74	M	CT	2	28	5	7	19
*P14*	54	M	CT	3	28	1	4	15
*P16*	49	F	CT	3	30	5	23	26
*P18*	69	M	CT	4	21	2	47	25
*P20*	69	F	CT	3	29	2	5	12
*P21*	56	F	CT	2	29	6	18	24
*P22*	69	M	CT	2	25	8	6	16
*P24*	62	F	CT	3	30	2	58	26
*P27*	68	F	CT	3	28	4	37	29

IT and CT refer to intervention therapy (Robot Gym) and control therapy, respectively. This table shows the baseline of different tests for each patient that concluded the protocol

The pilot trial recruitment stopped at a sample size of 10 in each group because both groups had the same sample size and other new patients who accomplish all the inclusion criteria were not available.

The mean age of the study participants per group is shown in Table 2. The inclusion criteria measurements made prior to the intervention to ensure study participant homogeneity were the following: the Mini-mental Scale Test (Table 3), the Geriatric Depression Scale Test (Table 4), and the Brunnstrom Test (Table 5). Although the RT group was significantly younger than the CT group (RT:44.1 ± 12.55 versus CT:64.1 ± 8.38; *p* <0.0006), both groups had comparable cognitive function assessed using the Mini-mental Scale Test (RT:28.5 ± 2.01 versus CT:27.6 ± 2.72; *p* <0.411), mental health assessed by the Geriatric Depression Scale (Table 2; *p* = 0.401) and initial motor function assessed by the Brunnstrom test (Table 3; *p* = 0.5724). The Geriatric Depression Scale measures mental health using a scale from 1 to 8 according to their emotional status. The Brunnstrom Test qualitatively classifies subjects using a scale from 1 to 5 according to their muscular movement impairment.

**Table 2:** Mean age of study participants according to group

Group	Age
Robotic (*n* = 10)	44.1 ± 12.55
Control (*n* = 10)	64.1 ± 8.38
*P* Value	0.00055

**Table 3:** Mean scores of pre-intervention mini-mental scale test results according to group

Group	Pre-intervention mini-mental scores
Robotic (*n* = 10)	28.5 ± 2.01
Control (*n* = 10)	27.6 ± 2.72
*P* Value	0.411

**Table 4:** Pre-intervention depression scale scores

Group	Pre-intervention depression scores									
	# of Patients given a score									
Possible depression scale scores	0	1	2	3	4	5	6	7	8	
Robotic	1	2	1	3	1	1	0	1	0	Sum of col. = 10
Control	0	2	3	0	1	2	1	0	1	Sum of col. = 10
Chi-Squared Value	8.33									
*P* Value	0.401									

The possible scores are the values the patients could archive. Thus, this test is qualitative. The numbers shown in the robotic and control rows are the number of patients that were given that possible score in its column pre-intervention. For example, one patient in the RT were given the score of “0” and no patient in CT was given the score of “0”

**Table 5:** Pre-intervention Brunnstrom test results

Group	Pre-intervention Brunnstrom Scores				
	# of Patients given a score				
Possible Scores	2	3	4	5	
Robotic	2	5	2	1	Sum of col. = 10
Control	4	5	1	0	Sum of col. = 10
Chi-Squared Value	2				
*P* Value	0.572				

As the Brunnstrom Test is a qualitative test, the numbers shown on the robotic and control rows represent the number of patients that were given the possible score in its column

### Upper extremity assessment results

The pre- and post-intervention scores for the Fugl-Meyer Upper Extremity Test (UE-FM) and Box and Block Test (BBT) are shown in Table 6. The change generated after the intervention is also shown. Both groups showed improvement with a UE-FM score that increases more than 4 points post-intervention (RT: 4.6 ± 3.89 and CT: 5.1 ± 4.72). No statistically significant differences in improvement were observed when comparing the two groups (*p* = 0.799), suggesting that each intervention was equally effective. Although not statistically significant, the RT group showed more improvement in the BBT scores than the CT group (RT: 2.2 ± 3.61 and CT: −0.3 ± 3.30; *p* = 0.124). Overall, there was improvement in both groups. Effect size calculations show that both RT group and CT group had a small effect (0.34 and 0.25, respectively) on UE-FM, whereas the RT group had higher effect on BBT outcomes (0.23) compared to the CT group (−0.03).

**Table 6:** Upper extremity test results

Upper Limb Scores	Group	Pre	Post	Effect size (Cohen’s d)	Change
Fugl-Meyer (UE-FM) Max score is 66	Robotic (*n* = 10)	23 ± 12.59	27.6 ± 14.70	0.34	4.6 ± 3.89
	Control (*n* = 10)	22 ± 19.17	27.1 ± 22.03	0.25	5.1 ± 4.72
	*P* Value	0.891	0.953		0.799
Box and Block (BBT)	Robotic (*n* = 10)	3 ± 8.46	5.3 ± 11.28	*0.23*	2.2 ± 3.61
	Control (*n* = 10)	4.7 ± 9.45	4.4 ± 7.26	−0.03	−0.3 ± 3.30
	*P* Value	0.676	0.834		0.124

The Fugl-Meyer (UE-FM) maximum score is 66.BBT is in # of blocks in 1 min

The results of the Functional Test for the upper extremity (UE-FT) are shown in Table 7. The score row shows the possible UE-FT score where the patient can be assigned. As shown with the UE-FM and BBT, both groups improved. The change column score displays how many subjects changed score levels after the intervention. For example, in the RT group, one subject decreased two levels, two subjects improved one level, two subjects improved three levels, and five subjects did not change. No statistically significant differences were found when comparing the RT and CT group pre- and post-intervention.

**Table 7:** Functional test scores

Group	Pre							Post							Change				
Possible FT Scores	1	2	4	5	6	7		1	2	4	5	6	7		−2	0	1	2	3
Robotic	0	6	3	0	1	0	#10	0	5	0	4	1	0	#10	1	5	2	0	2
Control	3	3	2	1	0	1	#10	2	4	1	1	0	2	#10	0	7	2	1	0
Chi-Squared	8.33							8.05							4.33				
*P* Value	0.401							0.089							0.362				

The possible score row shows the possible values a patient could achieve in this test

### Lower extremity assessment results

The clinical change results for the lower extremity assessment using Fugl-Meyer Lower Extremity Test (LE-FM), 6-minute Walk Test (6MinWT), 10-meter Walk Test (10MtsWT) and Timed Up and Go Test (TUG) are shown Table 8. When comparing the pre-intervention LE-FM, no significant difference was observed between the two groups (RT: 23.1 ± 6.37 and CT: 20.1 ± 5.78; *p* = 0.284). After the intervention, both groups showed improvement, with the RT group presenting a significantly greater improvement compared to the CT group (RT: 26.4 ± 4.70 and CT: 20.6 ± 6.41; *p* = 0.033). The change in the RT group was on average of 2.8 points greater than the CT group. Effect size calculations show that the intervention had a moderate effect (0.59) on LE-FM outcomes in the RT group and little to no effect in the CT group (0.08). This moderate effect was not observed in the 6MinWT, 10MtsWT or TUG functional outcomes.

**Table 8:** Lower extremity test results

Lower Limb Scores	Group	Pre	Post	Effect size (Cohen’s d)	Change
Fugl-Meyer (LE-FM) Max score is 34	Robotic (*n* = 10)	23.1 ± 6.37	26.4 ± 4.70	0.59	3.3 ± 3.59
	Control (*n* = 10)	20.1 ± 5.78	20.6 ± 6.41	0.08	0.5 ± 1.71
	*P* Value	0.284	*0.033		*0.035
6 Minute Walk Test (6MinWT) (meters)	Robotic (*n* = 10)	214.6 ± 118.46	228.1 ± 126.53	0.11	13.5 ± 35.96
	Control (*n* = 10)	105.5 ± 95.51	107.4 ± 92.42	0.02	1.8 ± 15.80
	*P* Value	*0.036	*0.025		0.226
10 Meter Walk Test (10MtsWT) (seconds)	Robotic (*n* = 10)	30.9 ± 37.25	26.7 ± 26.20	−0.13	−4.2 ± 13.75
	Control (*n* = 10)	71.9 ± 52.33	74.1 ± 62.25	0.04	2.1 ± 30.57
	*P* Value	0.058	*0.019		0.496
Timed up and Go (TUG) (seconds)	Robotic (*n* = 10)	34.9 ± 37.52	31.6 ± 33.05	−0.09	−3.3 ± 6.75
	Control (*n* = 10)	77.1 ± 54.97	78.8 ± 63.39	0.03	1.7 ± 19.21
	*P* Value	0.060	0.051		0.820

*p <0.05 is significant

The 6MinWT and 10MtsWT functional walking tests demonstrated the improvement seen. An increase in distance walked in 6MinWT indicates improvement, whereas a decrease in time in 10MtsWT indicates improvement. For the 6MinWT, the RT and CT group show a statistically significant difference pre- and post-intervention with both groups presenting progress in their performance. Due to the large variability within the groups, the changes seen in the RT group (13.5 ± 35.96) were not significantly greater than those in the CT group (1.8 ± 15.80). A large variability in individual performance was also observed in the 10MtsWT. Despite a large mean score difference, there were no statistically significant differences observed when comparing the groups pre-intervention; however, the post-intervention 10MtsWT times were statistically different when comparing the groups. The RT group improved with a reduced time to perform the task of 4 s, but the change differences in this group were not significant due to the large standard deviations seen in performance. The groups did not have statistically significant differences when comparing the pre-intervention TUG times (*p* = 0.06). A decrease in the TUG test time indicates improvement. The average time to TUG in the RT group decreased after the intervention, but the average time in the CT group slightly increased. However, the changes seen in these values were not significantly different.

### Cost comparison

Traditional therapy staff costs were calculated using salaries of therapists. The salary of a rehabilitation specialized therapist in the Mexican public health institution is reported to be around $235,344 MXN ($19,612 USD) per year. It was assumed that the initial cost of the existing traditional therapy equipment, which is about 7,000 USD, is irrelevant since our intention is to augment the existing rehabilitation centers with robotic therapy. As such, only a maintenance and consumables fee per year was added to salaries. For the typical standard of care at CREE, and in fulfillment with Mexican therapy guideline, a therapist can attend up to four patients in a period of time of 2 h, which translates into 16 patients in a full 8-h work day. For the 278 working days at CREE this translates to 4448 patients in a year. For the control group in this protocol where standard of care was time-matched to the robot gym, therapists treated one patient for 2 h. This would equate to four patients per day in a full-time shift of 8 h and, for the 278 working days at CREE, about 1112 patients per year.

For the robot gym therapy numbers the staff cost for a rehabilitation specialized therapist was assumed to be the same as in the traditional therapy. The robot gym equipment was acquired in USA and imported to Mexico. The full cost of the robotic equipment (adding transportation and importation costs) is $432,592.4 MXN, to be settled within 2 years with annual payments of $216,296 MXN (approximately $18,024 USD at that time). A 25 % overhead was added into the numerator of the equation for maintenance and consumables, giving us $108,148 MXN. The number of patients seen in a session was dictated by the gym design. The design of the Robot Gym allows six patients to be supervised and receive 2 h of therapy, which translates in 24 patients a day, in a full-time shift of 8 h and, for the 278 working days at CREE, about 6672 patients per year.

Table 9 summarizes the variables these results. Traditional therapy, which consisted of the time-matched standard of care where patients received 2 h of therapy, was estimated to have therapy cost of $230.52 MXN ($19.21 USD) per session with the labor ratio of 1 to 1112 per year. Traditional therapy, which consisted of the standard of care where patients received 30 min of therapy per session, was estimated to have a therapy cost of $57.63 MXN ($4.80 USD) with the labor ratio of 1 to 4448. In contrast, the robotic therapy group, the therapy cost would be $83.90 MXN ($6.99 USD) within the first 2 year and after this period of time, the net cost of the equipment will be liquidated and only the percentage reserved for the maintenance will remain, reducing the cost to an estimated cost for robotic therapy of $51.48 MXN ($4.29 USD) per session with a 2 h therapy session per patient. The labor ratio is 1 to 6672 per year.

**Table 9:** Therapies cost analysis

	2 h Therapy		30 min Therapy	
	Treated patients per year	Cost per therapy	Treated patients per year	Cost per therapy
Traditional Therapy	1112	$19.21 USD	4448	$4.80 USD
Robot Gym Therapy	6672	$6.99 USD	N/A	N/A
Robot Gym Therapy After 2 years	6672	$4.29 USD	N/A	N/A

The table shows the cost of both therapies considering the number of patients that can be assessed giving a traditional 30 min session and a 2 h session

When treatment time is matched, the robot gym would allow six times more patients to be seen than time-matched standard of care for the control group and 1.5 times more than what is typically done in CREE. The robot gym therapy would deliver more time for therapy at the same cost as what is currently being done in the one-to-one traditional therapy. The cost of providing the time-matched traditional therapy would be 2.74 more expensive than using the robot gym in the first 2 years increasing to about 4.5 times more expensive thereafter.

## Discussion

Given the need for affordable alternatives to post-stroke rehabilitation and the lack of physical and occupational therapists in developing countries, we proposed and developed a Gymnasium for Robotic Rehabilitation. This system combines relatively low cost robotic therapy with intensive, task-specific therapies for both the lower and upper extremities. The Robot Gym introduces a combination of semi-independent therapies using a circuit-training concept and adding Functional Electrical Stimulation, cognitive, and gaming therapy that target activities of daily living. Circuit class or circuit training therapy uses different physical activity workstations combined in an intensive manner, involving more than two patients per therapist [72]. CCT provides a cost saving option reducing the staff to patient ratio and has been proven to be effective in improving walking capacity and mobility [73, 74]. Accumulating evidence supports that gaming therapy, the use of electronic games in rehabilitation settings, is more enjoyable and accepted by patients [75].

The data generated in this pilot study shows that the Robot Gym is a cost- and labor-efficient alternative for post-stroke rehabilitation. The results from the lower extremity tests (LE-FM, 6MinWT and 10MtsWT) support that the Robot Gym may be more effective or as effective as traditional physical and occupational therapy approaches. Subjects in the RT group significantly improved an average 3.3 points in the Fugl-Meyer Lower Extremity Test scores compared to the CT, which had an average improvement of 0.5 points (*p* = 0.033). The effect size in the LE-FM scores support that robotic therapy may be more effective than traditional therapy (0.59 versus 0.08). The same tendency seen in the Fugl-Meyer Lower Extremity Test, was observed in 10-meter Walk Test. The RT group walked faster, with a reduction of an average of 4.2 s, in contrast to the CT group, which took an average of 2.12 s longer to complete the 10 meter walk. In the post-intervention 6-minute Walk Test, the RT group walked an average 13.55 m more compared to the control group that walked an average 1.85 m more in the 6 min time frame. The statistically significant differences observed between the groups when comparing the post-intervention 10-meter Walk Test and 6-minute Walk Test results suggest that robotic therapy administered via the Robot Gym may be more efficient than traditional therapy. These results are supported by the evidence presented by Buschfort and colleagues [35] and Hesse and colleagues [26] showing the effectiveness of circuit training using robots in a rehabilitation hospital in Germany, a high income country. Our results support the use of robots in this manner and show that it has the potential to augment access issues in middle income countries such as Mexico.

Both intervention groups were functionally similar prior to the intervention according to the clinical test but not in age. All of the pre-intervention test results showed a no statistically significant differences between groups, with the exception of the 6-minute Walk Test. A statistically significant difference in pre- and post-intervention 6-minute Walk Test results between the groups with a *p*-value = 0.036 and a *p*-value = 0.0254, respectively. The difference between groups could be explained by the origin of this test. The 6-minute Walk Test was developed as a tool to measure functional cardiopulmonary capacity [67]. As the cardiopulmonary capacity declines with age [76, 77], the difference could be due the age difference between the groups. The subjects in the CT group were on average older than those in the RT group (*p*-value = 0.0005). The inclusion criteria tests that included the Mini-mental, the Brunnstrom Test, and the Geriatric Depression Scale tests were not significantly different between the groups. Therefore, both groups were considered functionally similar before the intervention. The age difference could be attributed to the size of the sample and the wide age range, 21 to 75 years old, in the inclusion criteria. Although functionally equivalent at onset of the treatment, the significant differences in age across study groups may limit the interpretation of the results. The effect of age should be considered in further studies to corroborate our findings. Other past studies have found that there is a small effect of age in stroke recovery, however this effect has been reported to be of minimal clinical relevance in other stroke rehabilitation studies [78, 79].

The results of the upper extremity tests suggest that the robotic intervention administered via the Robot Gym was as effective as traditional physical and occupational therapy approaches. The robotic and traditional therapy interventions had similar effect sizes (0.34 versus 0.25). No statistically significant differences were observed for the Fugl-Meyer Upper Extremity Test when comparing the groups pre- and post-intervention. The RT and the CT group improved an average of 4.6 points and 5.1 points, respectively. Similar results were observed for the Box and Block Test and Functional Test for the upper extremity, where both groups did not have statistically significant differences pre- and post-intervention. The Box and Block Test scores did indicate that the robotic therapy tended to have had a larger effect on grasping function than the conventional therapy. More subjects would need to be tested to confirm this trend. The conclusion that the robotic intervention was as effective as traditional physical and occupational therapy coincides with previous reports about robot-assisted therapy and the Theradrive system [27, 80–83]. These studies observed an improvement in functionality and motor recovery of upper extremity, but the results were not statistically different between groups.

Our study had a small sample size of chronic stroke survivors, which can be a limitation in our analyses, as it causes an imbalance on the mean age of the groups and should be taken into account. Therefore, we must be cautious in the interpretation of the results. Studies in low- and middle-income countries are often hampered by poor record keeping. In our study for example, patients are usually discharged after 3 months of therapy. In public health centers in particular, records are not kept up to date after patients are discharged. Because of this, our sample size was limited and the age range of the patients in the inclusion criteria had to be kept wide to allow for more patients to participate in the study. Despite this sample size limitation, the significance and effect sizes observed suggest that therapy administered via the Robot Gym is more or as valuable as traditional therapy. However, it is important to mention that we are only evaluating the short term effect of the therapy and not the long term effect.

The fact that the robotic gym therapy is cost-efficient with a favorable labor ratio could play an important role in the future of the implementation of this new concept. As for the initial investment in equipment, traditional therapy settings may include a therapeutic ultrasound, transcutaneous electrical nerve stimulation, laser therapy, and mechanotherapy equipment. The costs can vary depending on the complexity of the equipment, but an approximate cost of 7,000 USD is common. The cost of building the Robot Gym, including the commercially available equipment and the low-cost custom built equipment, was approximately 45,000 USD paid over 2 years. Even though the Robot Gym was designed to be low cost, it exceeds the initial cost of a traditional rehabilitation setting, which is usually the case with the use of actual robots and has been reported in the literature [32]. However, viewing the results of the cost analysis comparison of both therapies, we can conclude that, despite the initially higher cost, in the near and long term, the robot gym is more cost-effective than the traditional one-to-one therapies.

One of the main problems in public rehabilitation settings in low and middle-income countries is the availability of physical therapists and rehabilitation doctors [84]. It is common to find a low density of health professionals in developing countries such as Mexico where, for example, the ratio of health workers to patients is approximately 5.14 times lower than in the US [18]. The physical therapist in a public setting in Mexico usually spends 30–40 min per patient, which allows the therapist to treat 10 to 16 patients in an 8-h working day. With the help of the Robot Gym, a therapist could treat six patients which could result in a maximum of 24 patients receiving a 2 h therapy in an 8-h shift. The initial investment to set up a Robot Gym is 4 times higher than the investment required to set-up a traditional therapy setting; however, as showed in results, the initial expenditure is compensated by the increased number of patients seen per therapist when providing robotic gym therapy. Group therapy could also offer a similar labor efficient to the robotic therapy but these type of therapies were not considered in this study as it is not the standard therapy and we did not find any public health clinic in the region offering these type of therapies.

In the future we anticipate further revising this robotic gym concept to be even more cost-effective and more applicable to other low- and middle-income countries.

## Conclusion

There is an increased need for effective and affordable rehabilitation approaches that maximize functional recovery when stroke survivors are released from the hospital to the clinic. With the Robot Gym, we can improve rehabilitation capacity and leverage technology to reduce rehabilitation costs and maximize the utilization of therapy personnel resources. This concept allows the patient to receive comprehensive, intensive periods of therapy to improve their chances of recovery and expedite their return to their social and productive environment. The results of this pilot study showed that stroke patients that received therapy using the Robot Gym had improved functionality supporting that technology-assisted rehabilitation is more or as efficient as traditional therapy. Furthermore, the Robot Gym is a more cost- and labor-efficient therapy option since only one therapist is needed to supervise six patients, making it a feasible option for countries with scarce clinical resources and funding. However, the effect that working in groups with gaming therapy in the Robot Gym setting might have on patient motivation needs to be evaluated. A study with a larger sample may help to further corroborate our findings. The transferability of the Robot Gym to health care settings could be further explored by applying the concepts used in this study to evaluate the effectiveness of this type of therapy in the rehabilitation of individuals with a wider range of functional disabilities.

## Abbreviations

10MtsWT: 10 Meter walk test
6MinWT: 6 Minute walk test
ADLs: Activities of daily living
BBT: Box and block test
CCT: Circuit class or circuit training therapy
CNS: Central nervous system
CT: Control group
DIF: Desarrollo Integral de la Familia
FES: Functional electrical stimulation
IRB: Institutional Review Board
LE-FM: Fugl-Meyer lower extremity
PHI: Protected health information
Robot Gym: Gymnasium for robotic rehabilitation
RT: Robot intervention group
TENs: Transcutaneous electrical nerve stimulation
TUG: Timed up and go test
UE-FM: Fugl-Meyer upper extremity
UE-FT: Functional test for the upper extremity
